# Supplementary material for: Stress Corrosion Cracking Behavior of Fine-Grained Al5083 Alloys Processed by Equal-Channel Angular Pressing (ECAP)
Source: Molecules. 2021 Dec 15;26(24):7608. doi: 10.3390/molecules26247608 (PMC8707193; doi:10.3390/molecules26247608)
Supplement: Supplementary file 1 [file molecules-26-07608-s001.zip › molecules-1423510-SI.pdf]

Supplementary Material

Stress Corrosion Cracking Behavior of Fine-Grained Al5083 Alloys Processed by Equal-Channel Angular Pressing (ECAP)

Asiful H. Seikh [1,*], Muneer Baig [1,2] , Ateekh Ur Rehman [3] and Faraz H Hashmi [4]and Jabair A. Mohammed [1]

[1] Centre of Excellence for Research in Engineering Materials, Deanship of Scientific Research, King Saud University, P.O. Box-800, Riyadh 11421, Saudi Arabia; A jmohammed@KSU.EDU.SA (J.A.M. and .S.A.H.)
[2] Engineering Management Department, College of Engineering, Prince Sultan University, P.O. Box-66833, Riyadh 12435, Saudi Arabia; mbaig@psu.edu.sa (M.B.)
[3] Department of Industrial Engineering, College of Engineering, King Saud University, P.O. Box 800, Riyadh 11421, Saudi Arabia; arehman@ksu.edu.sa (A.U.R)
[4] Department of Mechanical Engineering, College of Engineering, King Saud University, P.O. Box 800, Riyadh 11421, Saudi Arabia; 439106627@student.ksu.edu.sa (F.H.H.)
* Correspondence: aseikh@ksu.edu.sa

Supplementary Figure: XRD Graphs used for phase identification.

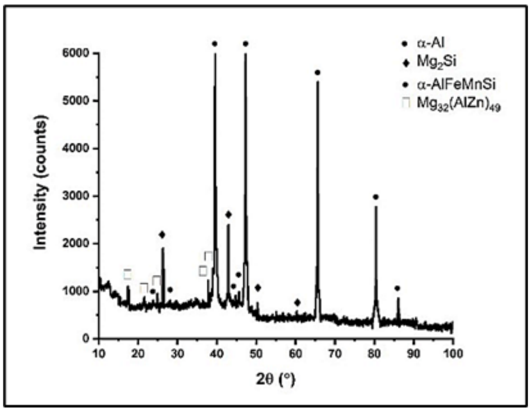

(a)

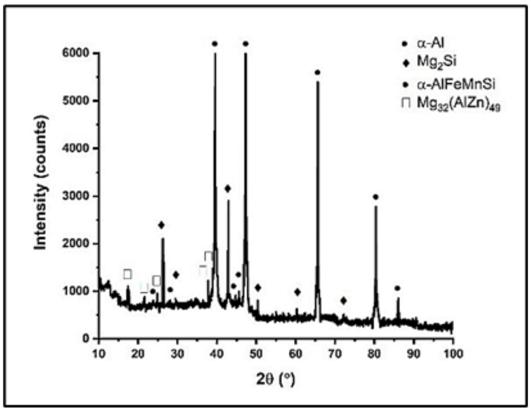

(b)

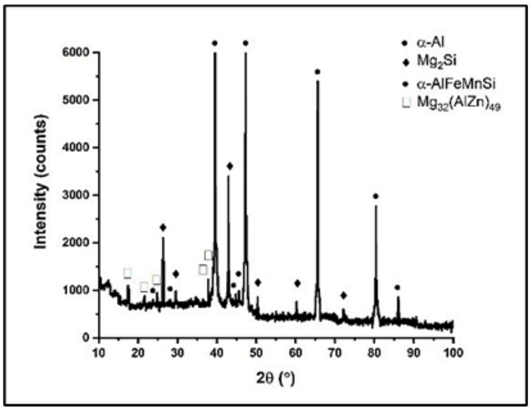

(c)

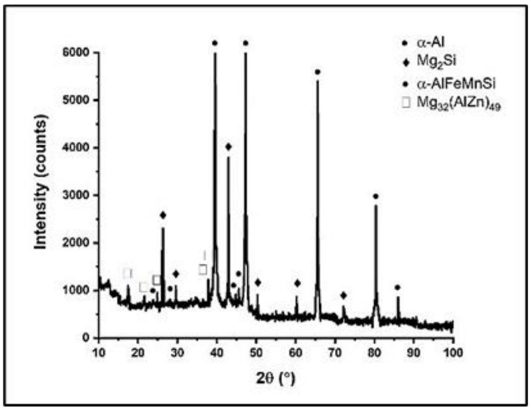

(d)

XRD graphs of Al5083 alloy samples: (**a**) as-received; (**b**) ECAP 1st pass; (**c**) 2nd pass; (**d**) 3rd pass.

Explanation: How to determine the "% error"

Each test was conducted three times using software and the standard deviation method to calculate the error. The correlation coefficient (R) is a statistical tool that provides information on the strength of a linear relationship between experimental and predicted values. The average absolute error is a quantity used to measure how close the prediction values are to the experimental ones.

Reference:

1. Desu, R.K.; Krishnamurthy, H.N.; Balu, A.; Gupta, A.K.; Singh, S.K. Mechanical properties of Austenitic Stainless Steel 304L and 316L at elevated temperatures. *Journal of Materials Research and Technology*, **2015**, *5*, 13–20, doi:10.1016/j.jmrt.2015.04.001.
